# The role of the meningeal lymphatic system in local meningeal inflammation and trigeminal nociception

**DOI:** 10.1038/s41598-022-12540-7

**Published:** 2022-05-25

**Authors:** Nikita Mikhailov, Anaïs Virenque, Kseniia Koroleva, Elisa Eme-Scolan, Matei Teleman, Ali Abdollahzadeh, Raisa Giniatullina, Oleg Gafurov, Georgii Krivoshein, Tarja Malm, Riikka H. Hämäläinen, Alejandra Sierra, Jussi Tohka, Rejane Rua, Francesco M. Noe, Rashid Giniatullin

**Affiliations:** 1 A. I. Virtanen Institute for Molecular Sciences, University of Eastern Finland, 70211 Kuopio, Finland; 2 HiLIFE-Neuroscience Center, Helsinki University, Haartmaninkatu 8, 00290 Helsinki, Finland; 3 Laboratory of Neurobiology, Kazan Federal University, Kazan, Russian Federation 420008; 4 Aix Marseille University, INSERM, CNRS, Centre d’Immunologie de Marseille Luminy, 13288 Marseille, France

**Keywords:** Diseases of the nervous system, Neuroimmunology, Molecular neuroscience

## Abstract

A system of lymphatic vessels has been recently characterized in the meninges, with a postulated role in ‘cleaning’ the brain via cerebral fluid drainage. As meninges are the origin site of migraine pain, we hypothesized that malfunctioning of the lymphatic system should affect the local trigeminal nociception. To test this hypothesis, we studied nociceptive and inflammatory mechanisms in the hemiskull preparations (containing the meninges) of K14-VEGFR3-Ig (K14) mice lacking the meningeal lymphatic system. We recorded the spiking activity of meningeal afferents and estimated the local mast cells population, calcitonin gene-related peptide (CGRP) and cytokine levels as well as the dural trigeminal innervation in freshly-isolated hemiskull preparations from K14-VEGFR3-Ig (K14) or wild type C57BL/6 mice (WT). Spiking activity data have been confirmed in an acquired model of meningeal lymphatic dysfunction (AAV-mVEGFR3(1–4)Ig induced lymphatic ablation). We found that levels of the pro-inflammatory cytokine IL12-p70 and CGRP, implicated in migraine, were reduced in the meninges of K14 mice, while the levels of the mast cell activator MCP-1 were increased. The other migraine-related pro-inflammatory cytokines (basal and stimulated), did not differ between the two genotypes. The patterns of trigeminal innervation in meninges remained unchanged and we did not observe alterations in basal or ATP-induced nociceptive firing in the meningeal afferents associated with meningeal lymphatic dysfunction. In summary, the lack of meningeal lymphatic system is associated with a new balance between pro- and anti-migraine mediators but does not directly trigger meningeal nociceptive state.

## Introduction

Migraine is a complex neurological disorder, with pain signalling likely originating from the meninges^1–3^ but the mechanisms determining the predisposition or development of migraine pain remain largely unclear. Meninges are densely innervated by somatic trigeminal^4,5^ and parasympathetic nerves^6^, which run along and in contact with local meningeal blood vessels^7^. It has been proposed that the so-called sterile neurogenic inflammation^8,9^ in the meninges is an important contributor to migraine mechanisms. The initial step in neurogenic inflammation is the activation of the trigeminal nociceptive system in meninges^10^. Indeed, trigeminal neurons release the calcitonin gene-related neuro-peptide (CGRP), which activates dural mast cells^11^. This key migraine related neuropeptide also promotes sensitization of trigeminal afferents^12,13^, and induces local vasodilation^14^. Vasodilation, in turn, can activate mechanosensitive receptors, expressed in trigeminal nerves within the meninges^15^. Trigeminal neurons can also be directly activated and sensitized by mast cells-secreted serotonin^16^, and by extracellular ATP, which is released from endothelial and mast cells, as well as from neurons, upon stimulation of the migraine mediator CGRP^17–19^. Finally, the release of acetylcholine (ACh) by parasympathetic fibers results in meningeal mast cell degranulation^20^, which content can directly activate the trigeminal nociceptive system^16^. In addition, endothelial^21^ and mast cells^22^ release various pro-inflammatory cytokines as potential contributors to local inflammation.

The presence of these diverse biologically active endogenous agents in the meninges suggests the existence of regulatory mechanisms, which limit the local level of pro-inflammatory mediators to minimize the activity of nociceptive fibers in normal conditions.

The recently characterized meningeal lymphatic vessels (mLVs)^23–25^ has been suggested to play a role in the clearance of the central nervous system (CNS) from toxic molecules, and their dysfunction to be involved in the pathogenesis of different neurodegenerative diseases^26–30^. However, the role of these vessels has not been so far evaluated in the context of migraine pathology.

Here we hypothesized that, due to their location coinciding with the origin site of migraine pain, meningeal lymphatics can control the local nociceptive state, while their malfunctioning can contribute to the pathology of migraine. To test this hypothesis, we assessed how developmental defects in the mLVs affect meningeal innervation, the population of resident mast cells, the production of migraine mediators (i.e., CGRP and pro-inflammatory cytokines), and the nociceptive spiking activity (both spontaneous and ATP-induced) in meningeal nerves. Our data demonstrate that, although functional defects in the meningeal lymphatics result in changes in the levels of migraine mediators, there is no direct involvement of these vessels in migraine pathology.

## Methods and materials

### Ethical statement

All procedures with animals were conducted in accordance with the European Community Council Directive 2010/63/EU and the ARRIVE guidelines. Experimental protocols involving the usage of animals were approved by the Animal Care and Use Committee of the University of Eastern Finland (license EKS-004-2014) and by the National Animal Experiment Board (ESAVI-2017-008787).

### K14-VEGFR3-Ig animals lacking meningeal lymphatics from the utero

We used adult (30–40 g) male and female transgenic K14-VEGFR3-Ig (K14) mice lacking the meningeal lymphatic system^24,31^ and wild-type C57BL/6 J littermate mice (WT). Total number of animals used for this study was n = 55 WT and n = 42 K14 animals. Animals were housed at the Lab Animal Center of the University of Eastern Finland under following housing conditions: 12 h dark/light cycle, grouped housing, given ad libitum access to food and water, 22 °C ambient temperature.

### Ablation of meningeal lymphatics in adult animals

Meningeal lymphatic ablation was achieved by systemic injection of an adeno-associated viral (AAV) vector expressing the human recombinant VEGFR3-1-4 fragment (VEGFR3-1-4Ig), as previously reported^23^. Adult C57BL/6 J male and female mice (3–4 month old) were injected with the AAV-mVEGFR3(1–4)Ig or the control vector AAV-mVEGFR3(4–7)Ig^23^. AAV-mVEGFR3(1–4)Ig encodes the ligand binding domains 1–4 of VEGFR3, fused to the IgG Fc domain: this molecule binds to both VEGF-C/D, inhibiting their binding to the VEGFR3, which is essential for lymphatic vessels formation and maintenance^31,32^. AAV-mVEGFR3(1–4)Ig and AAV-mVEGFR3(4–7)Ig mice were injected with a single i.p. dose of 10^12^ viral particles (in 200 µl sterile PBS) of the recombinant vector. Animals were euthanized and used for electrophysiology experiments 1 month after virus injection, when meningeal lymphatic vessels have been ablated^33^.

### Hemiskull preparation for ex vivo experiments

Transgenic K14 animals present diffuse defects in the lymphatic system, which involve several organs and structures. Thus, in order to study the specific contribution of the meningeal lymphatics to the activation of trigeminal nociception, and to avoid confounding factors, we used the ex-vivo hemiskull preparations, which preserve meninges and the meningeal trigeminal innervation^5,34^. This preparation allows us to directly examine the nociception mediators in the trigeminovascular system (i.e., meningeal resident mast cell population, CGRP release, cytokine release and trigeminal nerve spiking activity). For electrophysiology and CGRP release assay, animals were euthanized using CO_2_ without further anaesthesia. For cytokine release assay and mast cell staining, animals were first anesthetized with intraperitoneal injection of 250 mg/kg tribromoethanol (Avertin, Sigma-Aldrich, USA) and then transcardially perfused with: (1) normal saline (6 min at 6 ml/min), followed by 4% paraformaldehyde (PFA) (20 min at 3 ml/min) (mast cell staining); or (2) normal saline only (4 min at 20 ml/min) (cytokine release assay).

After decapitation, skin, muscles, and connective tissues were carefully removed. Thereafter, hemiskulls were isolated by cutting the skull along the sagittal axis and removing brain hemispheres with particular care to leave meninges and trigeminal innervation untouched. Two hemiskull preparations were obtained from each animal.

### Mast cell staining

Hemiskulls of WT and K14 animals were prepared as described above, washed three times for 20 min in PBS (phosphate buffered saline), and stained for 60 min with 0.1% toluidine blue. Stained meninges were immediately imaged, using a Zeiss Axio ZoomV16 stereomicroscope (Carl Zeiss AG, Germany), at 30× magnification. We imaged two identical regions-of-interest (ROIs) from each hemiskull: one including the superior sagittal sinus (SSS; at the border of the image) and the adjoining bottom one partially overlapping (approximately 10% of area) with the previous ROI. Size of each ROI was 3.7 × 2.4 mm^2^. In total, for each hemiskull we evaluated a 4 mm^2^ area, excluding edges, covering the region included between the middle meningeal artery and the sinus transversis. Using FIJI (ImageJ, National Institute of Health, USA), images were processed to detect mast cells using “Find Maxima” with the following parameters: noise tolerance 35, “Edge maxima” excluded. The positions (X,Y) of mast cells were then exported and counted in each quadrat (ROI = 5 × 5 quadrats) using Spatstat. The distribution of mast cells counts per quadrat was tested for normality and the associated *p* value was indicated for each mouse. Moreover, the Ripley k-function was used in combination with Monte-Carlo simulation to test for random distribution, using 100 simulations.

### CGRP release assay

CGRP release was assessed on hemiskull preparations of WT and K14 animals as previously described^15^. We used a CGRP enzyme immunoassay kit (EIA kit, SPI Bio, France) to measure CGRP release from hemiskull preparations. The levels of CGRP were assessed under control and KCl-treated conditions.

First, fresh hemiskull preparations from WT and K14 animals were recovered for 40 min in artificial cerebrospinal fluid (aCSF in mM: 119 NaCl, 30 NaHCO_3_, 11 glucose, 2.5 KCl, 1 MgCl_2_, 1 NaH_2_PO_4_, 2 CaCl_2_, adjusted pH level = 7.4) at room temperature (RT). Then, hemiskulls were washed four times with 150 µl aCSF for 15 min at 37 °C to stabilize the basal condition. Samples of aCSF (100 µl each) from the last two washes were collected to assess the basal level of CGRP (control conditions). After the last wash, hemiskulls were bathed with an aCSF solution containing 30 mM KCl for 15 min. Thereafter, a 100 µl sample was collected to assess CGRP under KCl-treated condition.

Samples were snap frozen with liquid nitrogen in tubes containing EIA buffer with peptidase inhibitors. Assay protocol was carried out in accordance with instructions of manufacturer. In brief, 100 μl of CGRP standard (for calibration curve) or sample were mixed with 100 μl of anti-CGRP AChE tracer in a well plate, preliminary washed 5 times with washing buffer. After incubating the plates at 4 °C for 16–20 h, supernatant was removed, and plates were washed 6 times with washing buffer. Finally, 200 μl of Ellman's reagent was added. Optical density was measured at 405 nm using microplate reader (Wallac VICTOR2, PerkinElmer, USA).

### Cytokine release assay

For cytokine release assay, we used hemiskull preparations from both WT and K14 mice. One hemiskull/animal was used to assess the release of cytokine under the control condition, while the contralateral was used to measure the release of cytokine under treatment condition (100 µM benzoyl ATP, BzATP). After the collection of the hemiskulls all procedures were performed at 37 °C. Hemiskulls were bathed in either aCSF or aCSF + BzATP (150 µl total volume) for 3.5 h. aCSF samples (50 µl) were taken to assess the level of cytokines in control (treated with aCSF only) or stimulated (aCSF + BzATP) conditions. The more stable ATP-analogue BzATP was used for the cytokine release assay, due to the long treatment time (3.5 h) and because of fast degradation of ATP^18^. The levels of aCSF in the preparations were monitored throughout the incubation period. If needed, we refilled the hemiskull preparation with the respective treatment solution to compensate for evaporation.

To assess the level of cytokines (IL-6, IL-10, MCP-1, TNFα, IFNγ, IL-12p70), we used the CBA Mouse Inflammation Kit (Cytometric Bead Array, BD Biosciences, New Jersey, USA). All procedures (sample and standard beads preparation) were performed following instructions provided by the manufacturer. Data were acquired on the CytoFLEX S cytometer (Beckman Coulter Inc., USA) and analyzed with the Flow Cytometric Analysis Program (FCAP) Array software (BD Biosciences, USA).

Due to the low concentration of IL-12p70 and IFNγ, in some samples it was not possible to quantify the exact concentration with the abovementioned method. Thus, for these specific cytokines final *n* was lower than the total number of animals used for the assay.

### Electrophysiology

Direct recording of action potentials from the trigeminal nerve afferents innervating meninges was conducted using a validated assay, as described earlier^5,34^. Hemiskulls of WT and K14 animals were placed in a perfusion chamber with a constant flow (6 ml/min) of oxygenated aCSF. In order to access the nervus spinosus of the mandibular branch of the trigeminal nerve, a small incision in the dura mater was made, using a 27G needle. To expose free-floating end of the nerve, the nervus spinosus was cut. Next, the nervus spinosus was sucked into a glass recording electrode filled with aCSF. Recording of spontaneous and stimulated activity was conducted with the same electrode. We recorded 10 min of spontaneous activity to get a baseline and to assess the difference in basal activity between WT and K14 animals. Thereafter, we applied 100 µM ATP for 10 min to stimulate nociceptive firing in the trigeminal nerve^35^, followed by 10 min of washout. Recordings were acquired using a digital amplifier (ISO 80, WPI Inc., USA) with bandpass 300 Hz–3 kHz, gain 10,000. Signals were digitized at 125 kHz using a NIPCI 6221 data acquisition board (National Instruments, USA) and visualized with WinEDR software (Strathclyde University, UK). One hemiskull preparation from each animal has been used for electrophysiological recordings. All hemiskulls were used only once in single independent recordings.

### Meningeal nerve staining

For the meningeal nerve staining, meninges were gently isolated from PFA-fixed hemiskulls under a dissection microscope, as previously described^36^. Then, meninges were stained as described below. Meningeal whole-mount preparations were incubated with 1× PBS containing 2% normal goat serum (NGS), 1% bovine serum albumin (BSA), 0.1% Triton X-100, and 0.05% Tween 20 for 1 h at RT. Thereafter, meninges were incubated with rabbit anti-β-tubulin III (1:1000, Cat# T2200, Sigma-Aldrich, USA) overnight at 4 °C in PBS containing 1% BSA and 0.5% Triton X-100. Whole-mounts were washed in PBS at RT (3×) followed by incubation with Alexa Fluor 488-conjugated goat anti-rabbit IgG antibody (1:500, Cat# A11008, Invitrogen, USA) for 1 h at RT in PBS with 1% BSA and 0.5% Triton X-100. After further washing in PBS (3×) and in phosphate buffer (PB; 2×), meninges were mounted with VECTASHIELD mounting medium (Vector Laboratories, USA), including DAPI, and coverslipped.

Images were acquired using a Leica TCS SP8 X confocal system (Leica Microsystems, Germany) using the LAS X software. Images of the region of interest, adjacent to the middle meningeal artery, were acquired with a 20× objective with 0.75 numerical aperture (NA), with an in-plane pixel size of 1.14 × 1.14 µm^2^ and a z-step of 1 µm. The full images were created by merging 4 × 4 tile scans, covering a total area of 2034 × 2034 µm^2^, with a tissue thickness ranging from 23 to 46 µm.

### Axon segmentation and innervation analysis

Meningeal innervation has been analyzed using two different technical approaches by two independent laboratories. A semi-automated segmentation technique was developed to annotate axons and analyze the innervation in acquired 3D confocal microscopy images. We created 2D maximum intensity projection image along the direction of focal planes (i.e., the z-axis), and enhanced the axons applying a Frangi filter^37^. Each enhanced image (*P*) has been normalized to its range [0, 1], and its intensity complemented so that the axons appeared darker than their background. On image *P*, an experienced researcher (NM) defined each axon by marking it with two points: axonal centerline was defined by using the minimal path algorithm (which minimizes an functional energy on *P*) between the two points^38^. Given the axonal centerline, we used active contours^39^ to segment the underlying desired axon, initialized from the axonal centerline with the speed function 1 − *P*. For each defined axon, we measured the axonal diameter and the length of its centerline in 3D, as previously described^40^. For each meningeal tissue, we determined an innervation complexity value, by forming a graph from the segmented axons^41^, and reported the total axonal length and the number of axonal endpoints (Fig. 7B,C).

Separately, the meningeal innervation region-specific patterns have been analyzed. For the nerve coverage area calculation, images were manually cropped to exclude the border areas, and converted in 8-bit. An automatic local threshold was applied using the following parameters: Phansalkar, Radius: 40, Special parameters: 0, and the proportion of surface covered by the nerves on the ROI was extracted (% Area). For the calculation of the nerve coverage intensity, the mean fluorescence intensity of Tubb3 stained meninges was calculated (Mean gray value). For the computation of Gaussian distribution, Tubb3+ pixels were detected using Imaris software 9.6.0 with the following parameters of spot detection: 10.0 µm, background substraction, Quality > 500. X and Y positions were extracted and counted in each quadrat (ROI = 10 × 10 quadrats) using Spatstat. The distribution of Tubb3+ spots per quadrat was tested for normality and the associated *p* value was indicated for each mouse. Results are presented on Fig. 7D–F.

### Statistical analysis

We performed statistical analyses using the GraphPad Prism 8.4.2 software (GraphPad Software, California, USA). The data are presented as mean ± the standard error of the mean (for two-way ANOVA and Student’s t-test) or as median with interquartile range (for Mann–Whitney U test and general mixed effect model). For all analyses, statistical significance was defined at α = 0.05. Mast cell number, as well as axon segmentation and innervation, were analysed using Student’s t-test. CGRP release was analysed with repeated measures two-way ANOVA. Cytokine release assay was analysed by Mann–Whitney U test (vs. basal cytokine production), or by matched two-way ANOVA (vs. stimulated cytokine production). A general mixed effect model was used to evaluate differences in the electrophysiology data. Bonferroni correction was used to adjust *p* values in multiple comparison. In the specific cases where both hemiskulls from the same animal have been used for the same analysis (i.e., mast cell densities), results from left and right hemiskulls had been averaged. In every experiment n = number of biological replicates (i.e., animals). For each experiment, the specific statistical test applied is indicated in the figure legend.

## Results

### Mast cells in meninges of mice without lymphatic system

**Figure 1:**
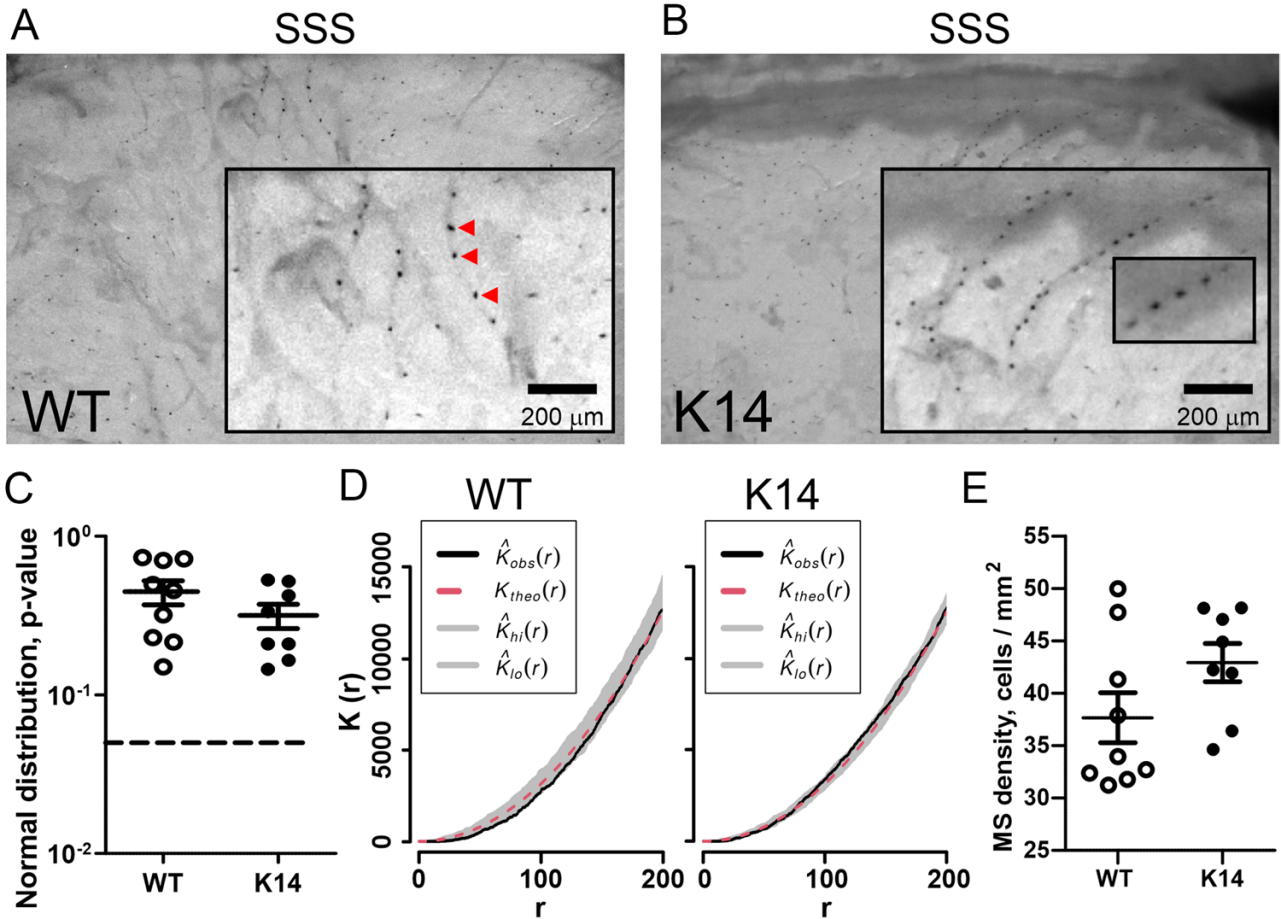
Dural mast cell population in WT and K14 mice. Mast cells localize in meninges of both WT (**A**) and K14 mice (**B**). Note the presence of mast cells (red triangles) aligned along putative meningeal blood vessels, afferent to the superior sagittal sinus (SSS, located at the top of the figure). (**C**) Mast cell normal distribution was tested using quadrat test, and the associated Gaussian *p* value was plotted for each mouse (WT n = 9, K14 n = 8; t(15) = 1.334, *p* = 0.2 by unpaired Student’s t-test). *p* values > 0.05 (dotted line) do not support the rejection of the hypothesis of Gaussian distribution. (**D**) Representative graphs showing the mast cell distribution, as calculated using the Ripley k-function. Of note, the function output is within the range of the Monte-Carlo envelope, which indicates random distribution. (**E**) Mast cell density (cells/mm^2^) was counted in WT and K14 animals. Transgenic animals show a non-significant increase in the number of mast cells (WT n = 9, K14 n = 8; t(15) = 1.717, *p* = 0.11 by unpaired Student’s t-test).

Meningeal mast cells have recently emerged as important players in the initiation of a migraine attack^16,42–44^. Since meningeal lymphatic vessels (mLVs) serve as important routes for circulation of immune cells within the CNS^25,45^, we expected that the lack of mLVs could affect the local population of mast cells within the meninges. Using the toluidine blue labelling, we quantified the number of resident mast cells in the meninges and characterized their distribution profile (Fig. 1A,B).

In both WT and K14 animals, we tested for normal distribution of mast cell in the meninges (Fig. 1C): in both genotypes, mast cells were equally normally distributed (*p* = 0.2), and their distribution pattern, as calculated using the Ripley k-function, was random (Fig. 1D). These data suggest that mLVs do not drive the specific distribution of mast cells in the meninges, and no spatial association exist between mast cell and lymphatic vessel location. However, a trend towards increased density of meningeal mast cells, although not significant, was found in K14 animals (38 ± 2 cells per mm^2^ in WT vs. 43 ± 2 cells per mm^2^ in K14; *p* = 0.11) (Fig. 1E).

### Cytokines release in meninges

It is generally accepted that migraine is associated with local sterile inflammation in meninges^10^. Dural immune cells can release local cytokines and pro-inflammatory mediators^19,42,44^, which promote meningeal inflammation and stimulate trigeminal afferents^16,17^. Therefore, we analysed the levels of the migraine-associated cytokines released from the hemiskull preparation of K14 and WT mice, in basal condition and after ATP stimulation.

**Figure 2:**
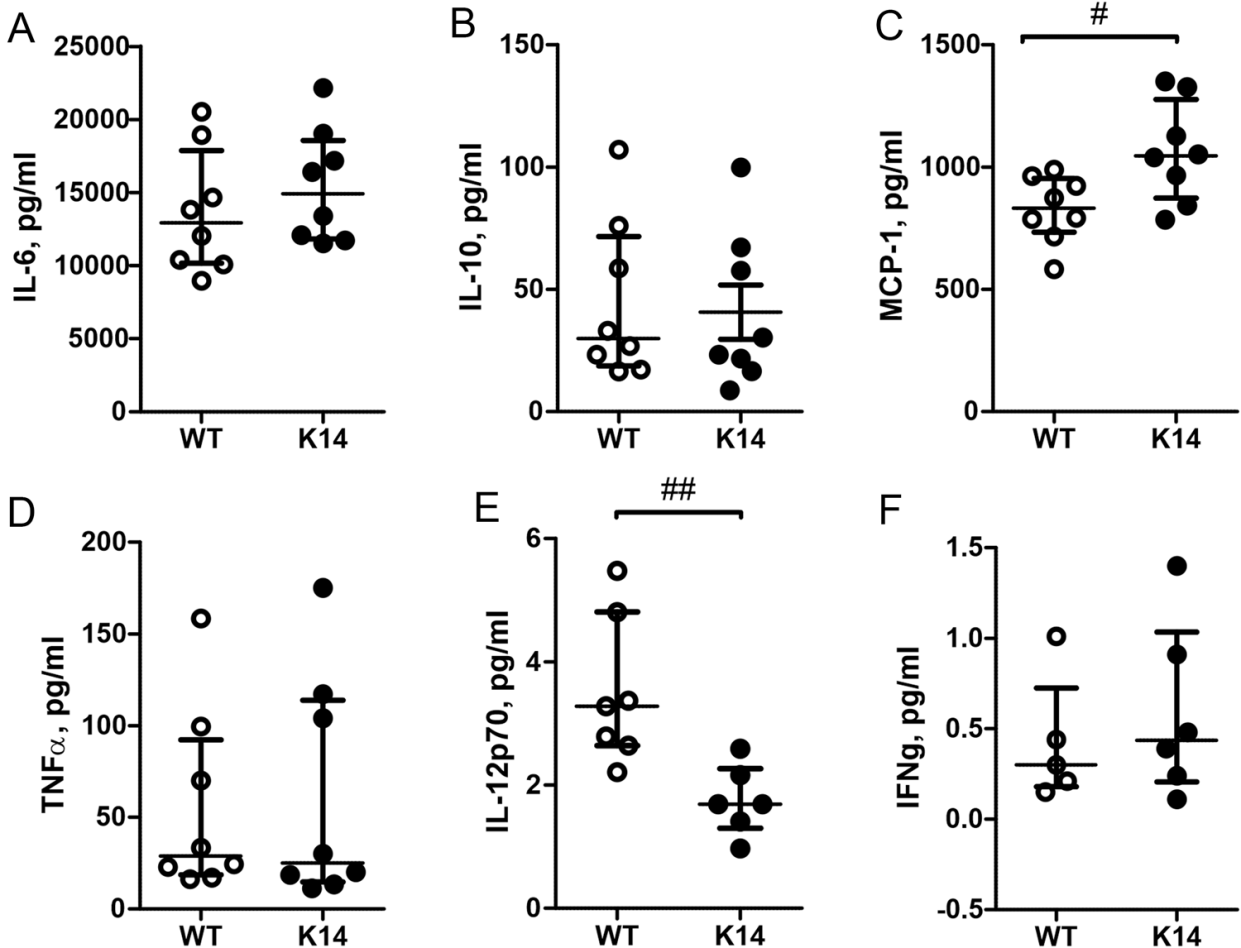
Basal cytokines release from meninges in the hemiskull preparation. Concentrations (pg/ml) of following cytokines are presented: IL-6 (**A**), IL-10 (**B**), MCP-1 (**C**), TNFα (**D**), IL-12p70 (**E**) and IFNγ (**F**). In K14 animals, we found a significant increase in the levels of MCP-1 (**C**), whereas the levels of IL-12p70 (**E**) were significantly lower compared to the one measured in WT littermates. No difference was found for the other measured cytokines. Number of replicates for IL-6, IL-10, MCP-1 and TNFα: n = 8/experimental group; for IL-12p70: WT n = 7 and K14 n = 6; for IFNγ: WT n = 5 and K14 n = 6. Mann–Whitney U test.

To this end, we measured the release levels of a panel of pro- and anti-inflammatory cytokines (Fig. 2A—IL-6, 2B—IL-10, 2C—MCP-1, 2D—TNFα, 2E—IL-12p70, and 2F—IFNγ) in basal conditions. We found that level of MCP-1 was significantly higher in K14 animals compared to their WT controls (829 ± 48 pg/ml in WT vs. 1061 ± 72 pg/ml in K14 respectively; n = 8 for each experimental condition; *p* = 0.028) (Fig. 2C). On the contrary, the basal level of IL-12p70 was significantly decreased in K14 animals compared to WT littermates (3.5 ± 0.5 pg/ml in WT vs. 1.8 ± 0.2 pg/ml in K14 respectively; WT n = 7, K14 n = 6; *p* = 0.002) (Fig. 2E). No differences in the levels of all the other measured cytokines were observed in basal conditions between the two genotypes.

**Figure 3:**
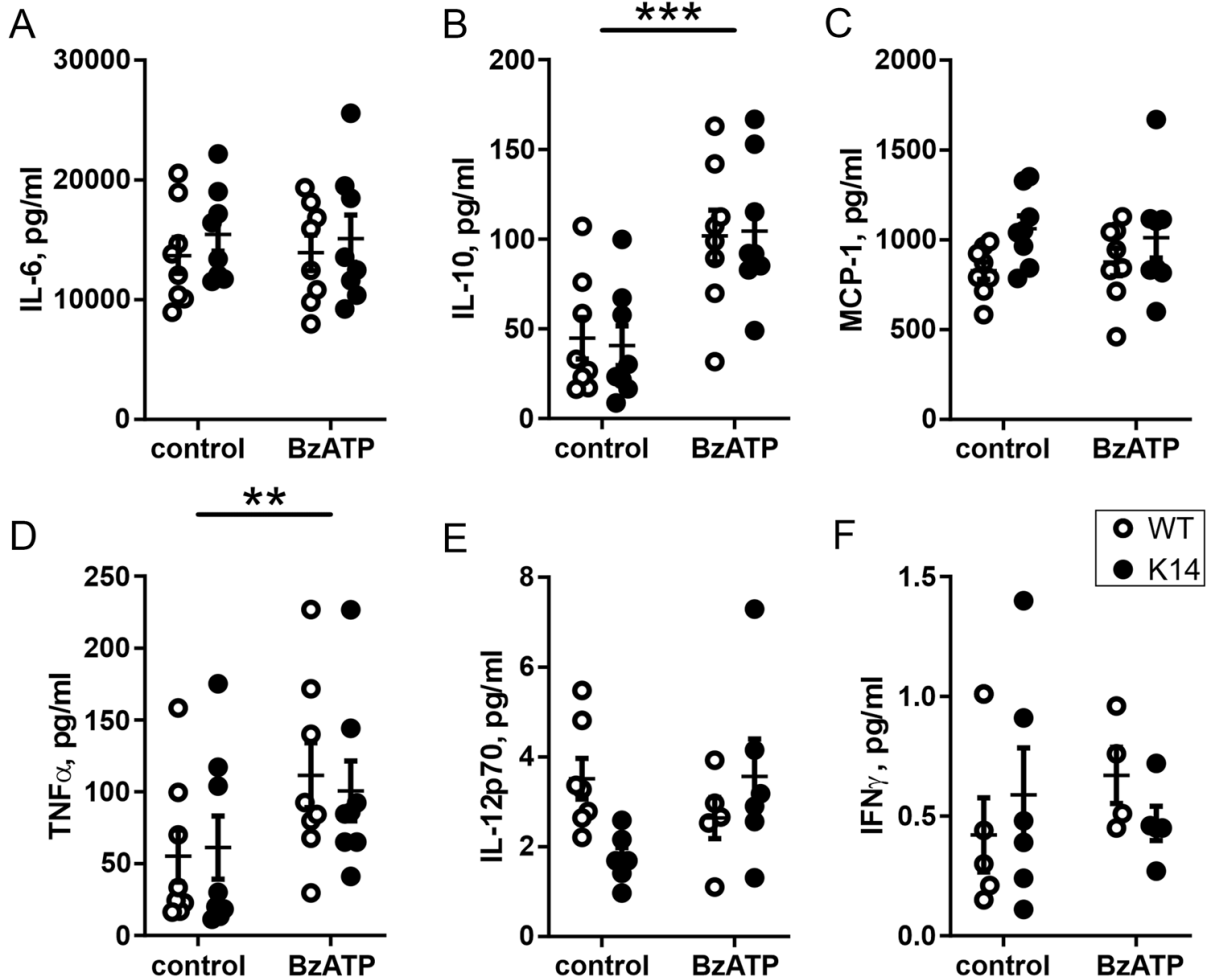
Cytokines release after BzATP stimulation. Scatter plots representing the concentrations of cytokines released from meningeal preparation after stimulation with BzATP: IL-6 (**A**), IL-10 (**B**), MCP-1 (**C**), TNFα (**D**), IL-12p70 (**E**) and IFNγ (**F**). BzATP induced an increase in the release of IL-10 (**B**) and TNFα (**D**) in both the genotypes and post-hoc comparisons revealed no differences in cytokines levels between WT and K14 preparations. Number of replicates for IL-6, IL-10, MCP-1, and TNFα: n = 8/experimental group; for IL-12p70: n = 7/6 (control) and n = 5/6 (BzATP) for WT and K14, respectively; for IFNγ: n = 5/6 (control) and n = 4/5 (BzATP) for WT and K14, respectively. ***p < 0.001 and **p < 0.01 (treatment effect) by matched two-way ANOVA with Bonferroni post-hoc test.

To mimic the inflammatory conditions contributing to migraine, we applied BzATP (a stable ATP analogue)^46^, which stimulates the pro-inflammatory P2X7 receptors, typically presented in the immune cells^47^, and measured the amount of released cytokines (IL-6, IL-10, MCP-1, TNFα, IL-12p70, and IFNγ) (Fig. 3A–F). Notably, the P2X7 receptor is also expressed in the dural mast cells^48,49^. Stimulation with 100 µM BzATP significantly increased the levels of IL-10 (treatment effect: *p* < 0.001) (Fig. 3B) and of TNFα (*p* = 0.003) (Fig. 3D), two mediators of migraine^50,51^. However, this pro-inflammatory increment did not differ between the WT and K14 mice. BzATP stimulation did not induce the secretion of the other measured cytokines, suggesting a selective sensitivity of the local inflammatory mechanisms to purinergic stimulation.

### Decreased CGRP release

**Figure 4:**
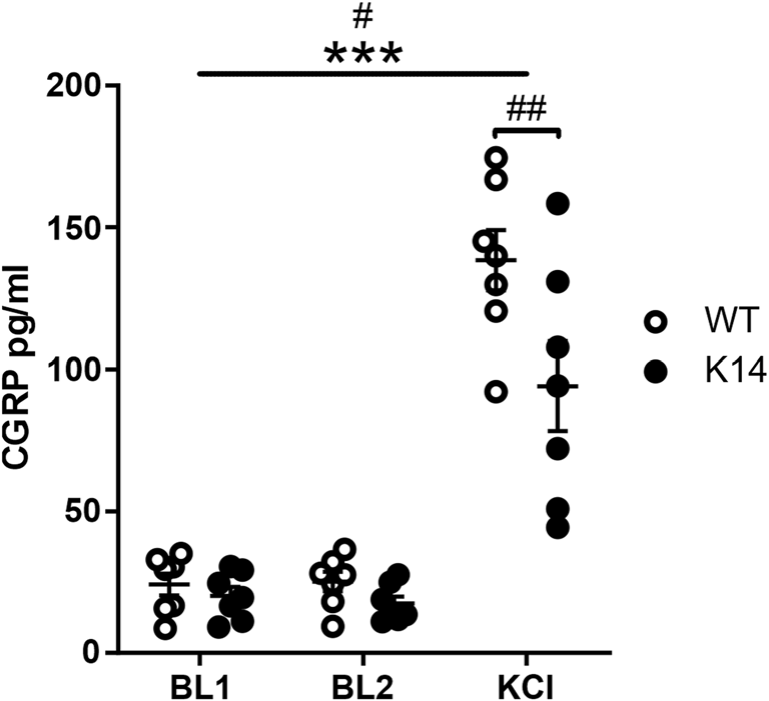
CGRP released from the meningeal trigeminal nerve endings in the hemiskull preparation. CGRP release was not altered in K14 hemiskulls under basal conditions. The application of 30 mM KCl triggered a significant increase in the CGRP level in both genotypes, and overall CGRP release was higher in WT animals. Post-hoc comparisons revealed that the concentration of the released CGRP under the stimulation was significantly lower in the hemiskulls lacking the lymphatic system compared to the WT preparations. (**B**) We propose two possible explanations for the altered CGRP release: (1) lower density of trigeminal afferents releasing CGRP, or (2) lower expression of CGRP. ***p < 0.001 (treatment effect); ^#^*p* = 0.03 (genotype effect); ^##^*p* = 0.002 (KCl-WT vs. KCl-K14) by matched two-way ANOVA with Bonferroni post-hoc test.

Since trigeminal neurons present in the meninges are capable of releasing CGRP, a main mediator of migraine pain and a promoter of mast cell degranulation during migraine attacks^3^, we measured the level of CGRP released in meninges in both basal and stimulated conditions (Fig. 4). Basal CGRP levels, as measured in two consecutive samples (baselines, BL), were similar in both genotypes (24 ± 4 pg/ml in WT-BL1 vs. 20 ± 3 pg/ml in K14-BL1, and 25 ± 3 pg/ml in WT-BL2 vs. 17 ± 2 pg/ml in K14-BL2; *p* > 0.99 by matched two-way ANOVA with Bonferroni post-hoc test). The application of 30 mM KCl to the meninges led to CGRP release through the stimulation of local nerve fibers (treatment effect BL vs. KCl: *p* < 0.001 by matched two-way ANOVA). Interestingly, CGRP release was significantly lower in K14 compared to WT hemiskulls (94 ± 16 pg/ml in K14-KCl vs.138 ± 11 pg/ml in WT-KCl; *p* = 0.002).

### Spontaneous and ATP-induced spiking activity in meningeal nerves

**Figure 5:**
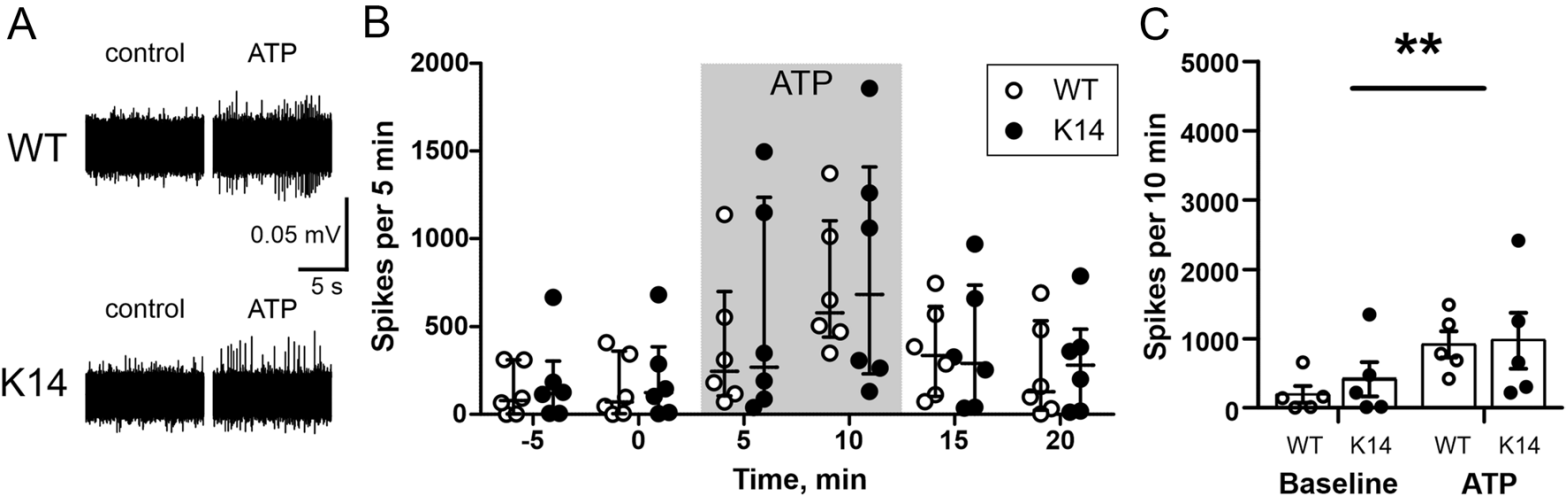
Direct spikes were recorded in WT and K14 hemiskull preparations under basal conditions and ATP stimulation. (**A**) Exemplificative traces of nociceptive spike recordings in WT and K14 animals under basal and ATP-stimulus conditions. (**B**) Number of nociceptive spikes recorded during 5 min bins (median with interquartile range) under basal conditions, during the application of 100 µM ATP, and during the following washout. (**C**) ATP induced a significant increase in nociception activity in both genotypes. **p = 0.0012 (baseline vs. ATP; n = 6/experimental group) by general mixed-effects model, but no difference was detected between WT and K14 animals (*p* = 0.711—genotype effect). Data presented as mean ± SEM.

One of the most direct approaches to study meningeal nociception, is the recording of spiking activity in trigeminal afferents^5^. Using this technique, we first tested the difference in spiking activity between WT animals and K14 animals having a developmental deficit in meningeal lymphatic vessels. First, we recorded the spiking activity in trigeminal nerve fibers from the hemiskull preparations under basal conditions. In WT animals, during the 10 min baseline, we recorded 193 ± 120 nociceptive spikes. Similarly, in K14 mice we recorded 413 ± 249 spikes per 10 min of baseline (*p* > 0.99 vs. WT). In order to mimic the conditions triggering the activation of nerve terminals in migraine, we applied 100 µM ATP, a powerful trigger of local nociception^17,18^ (Fig. 5A): Fig. 5B depicts the profile of nociceptive spike recording under the different experimental conditions (baseline, ATP-evoked activity and wash-out) in both WT and K14 mice. ATP application resulted in an identical increase in the nociceptive firing in both genotypes (WT: 917 ± 191; K14: 967 ± 404: *p* > 0.99), as recorded during the 10 min of ATP application (Fig. 5C), suggesting that a pre-existing mLVs dysfunction does not alter basal or triggered trigeminal nociception.

**Figure 6:**
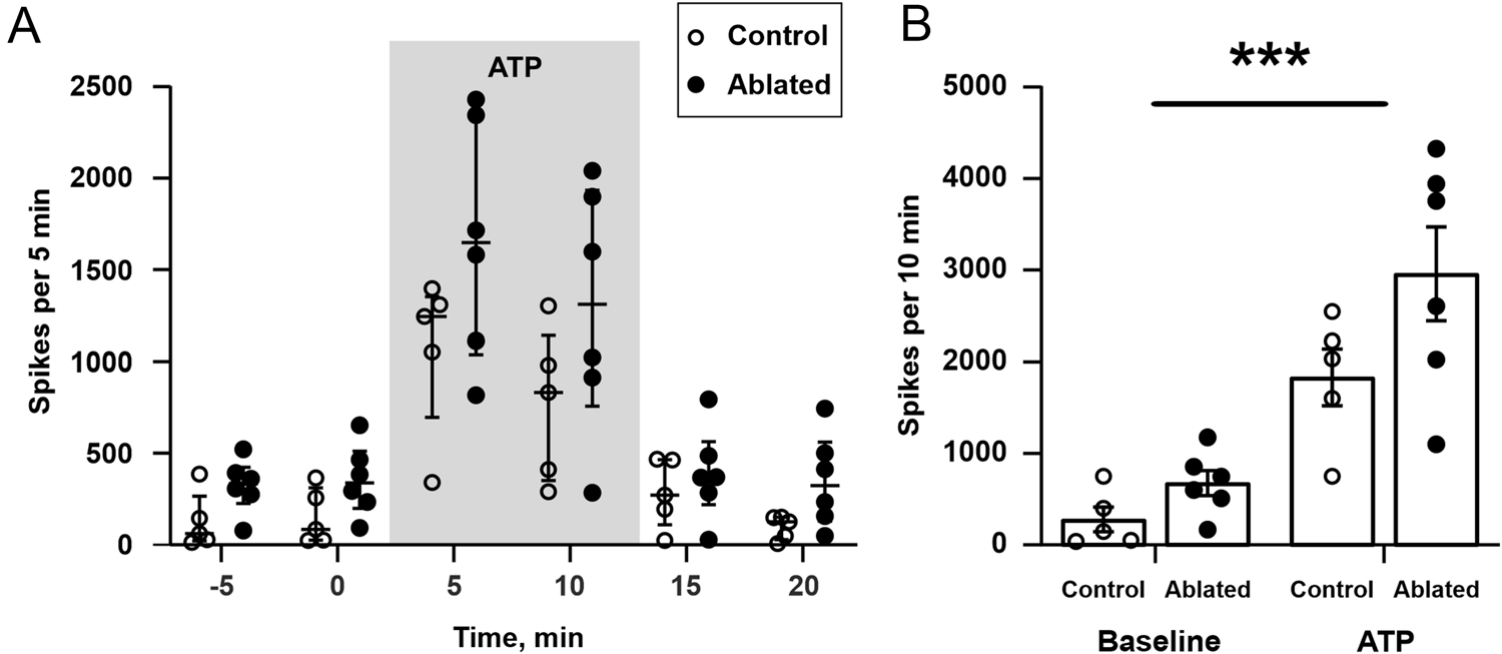
Direct spikes recording in control and in animals with AAV-ablated meningeal lymphatic system under basal conditions and ATP stimulation. (**A**) Number of nociceptive spikes recorded during 5 min bins (median with interquartile range) under basal conditions, during the application of 100 µM ATP, and during the following washout. (**B**) ATP induced a significant increase in nociception activity in both genotypes. ***p < 0.001 (baseline vs. ATP; n = 5/6 in control/ablated experimental group) by matched two-way ANOVA. Hemiskull preparations from mice with ablated lymphatics showed a non-significant trend for an increased response to ATP application (*p* = 0.077 vs. control). Data presented as mean ± SEM.

To validate the electrophysiology results obtained in K14 mice (which present a developmental deficit in the mLVs) and the main hypothesis that mLVs dysfunction does not alter trigeminal nociception, we ablated the meningeal lymphatics in adult mice and tested the induction of nociceptive spike activity as described before (Fig. 6A). Similar to results observed in K14 mice, the average number of spikes recorded per 10 min in control and ablated animals were similar under both baseline and ATP-treated conditions. During baseline, we recorded 279 ± 135 and 679 ± 139 spikes in the hemiskull preparations from control and ablated mice, respectively (*p* = 0.81). ATP exposure induced a significant and comparable increase in spiking activity in both experimental groups (1832 ± 311 and 2961 ± 514 spikes, in control and ablated hemiskull preparations; treatment effect, *p* < 0.001), with a non-significant trend for increased ATP-induced spiking activity in mice with ablated lymphatics (*p* = 0.055, control vs. ablated) (Fig. 6B).

### Unchanged patterns of meningeal innervation

**Figure 7:**
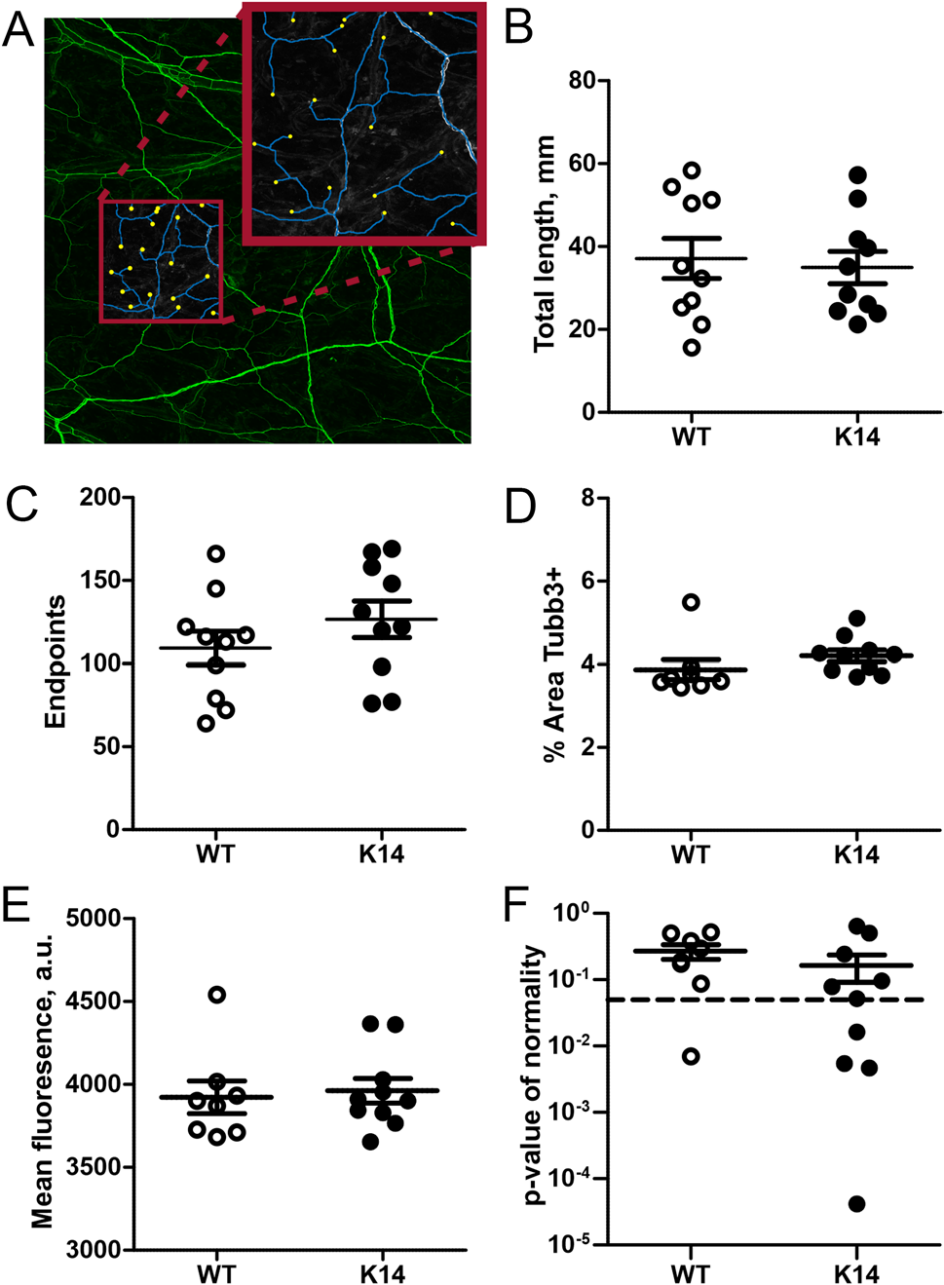
Segmentation analysis of meningeal innervation. (**A**) A representative image of meninges stained for β-tubulin III (green), segmented for morphological analyses. Blue lines represent axons, and yellow filled circles represent endpoints (i.e., CGRP-releasing trigeminal fiber terminal endings). (**B**) No differences in the total innervation length were observed between the two genotypes (*p* = 0.73 by unpaired Student’s t-test; n = 10/experimental group). (**C**) Similarly, the number of trigeminal fiber terminal endings was comparable in WT and K14 meninges (*p* = 0.26). (**D**–**F**) Analysis of the region-specific meningeal innervation patterns. No genotype differences were observed in the percentage of Tubb3 + area (*p* = 0.22, WT n = 8; K14 n = 10) (**D**), the intensity of Tubb3 fluorescence signal (*p* = 0.75) (**E**), and in the Gaussian distribution of nerves (*p* = 0.31). Data are presented as mean ± SEM.

The observed decrease in CGRP level can be due to a diminished meningeal innervation by nociceptive fiber^52^. To test this hypothesis, we evaluated the density of trigeminal innervation in the WT and K14 hemiskull preparations, by staining the meningeal preparations for the pan-neuronal marker β-tubulin III (Tubb3) (Fig. 7A)^53^. A semi-automated segmentation method has been applied to evaluate the total length of innervation (Fig. 7B), and number of endpoints (i.e., trigeminal fiber terminal endings, most of which are likely releasing CGRP) (Fig. 7C). We also evaluated region specific patterns (total stained area and intensity of staining) (Fig. 7D,E) and tested the normal distribution of the innervation (Fig. 7F). None of the analysed parameters showed a difference between the genotypes, suggesting that developmental dysfunctions in the meningeal lymphatic system do not affect the innervation of the meninges.

## Discussion

**Figure 8:**
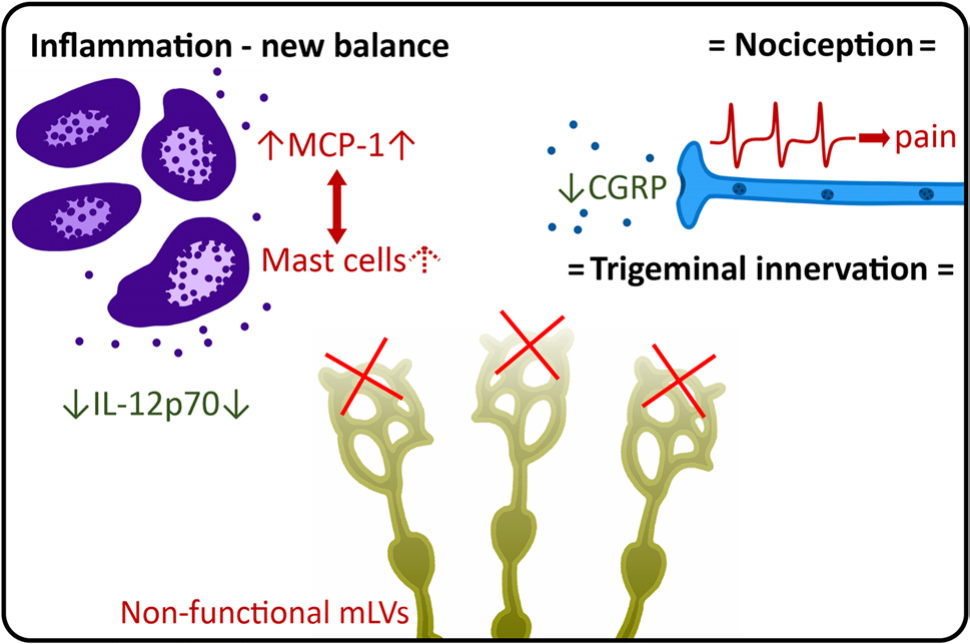
Summary of experimental findings. Dysfunctional meningeal lymphatic system triggers alterations in migraine mediator levels in the meninges. At basal conditions, the level of mast cell-activating cytokine MCP-1 is increased, supporting a possible increase in population of meningeal mast cells. In contrast, the release of CGRP and the level of pro-inflammatory IL-12p70 are decreased. However, these changes do not correspond to alterations in the trigeminal nociception. Finally, trigeminal innervation of the meninges was not changed, excluding a role of mLVs in this process. “Red” color indicates pro-inflammatory and pro-nociceptive changes; “Green” indicates anti-inflammatory and anti-nociceptive changes. Arrow up (↑) indicates an increase in the parameter, while arrow down (↓) indicates a decreased parameter. The equals sign (=) indicates unchanged parameters.

Our study evaluated for the first time, in the context of migraine pathology, the role of the meningeal lymphatic system in the regulation of the local neurochemical and cytokine profiles, as well as on the functional properties of meningeal nociception. We hypothesized that mLVs might participate in the induction of migraine pain signalling, which originates from the meningeal trigeminovascular system. Using a combination of different techniques, we characterized the inflammatory, cellular and nociceptive properties of ex vivo hemiskull preparation from WT mice and from mice presenting a developmental defect in the meningeal lymphatic system (K14). We found that the lack of meningeal lymphatics is associated with a specific profile of pro- and anti-nociceptive molecules. We observed a reduced release of the migraine mediator CGRP and of the pro-inflammatory cytokine IL-12p70. On the contrary, levels of MCP-1, which has been reported to be associated with migraine state in patients^54^ were increased. Mast cell counts, meningeal innervation, and trigeminal nociception did not show significant differences between WT and K14 mice. These data are summarized in Fig. 8.

Meninges comprise blood vessels, sensory nerve fibers and a high diversity of local immune cells^3,55,56^, and represent the main site for the generation of migraine pain^2,55^. In migraine-like conditions, meninges are involved in the so-called ‘neurogenic inflammation’, which is induced and maintained by the neuropeptides (e.g. CGRP) released from the trigeminal nerve fibers^57,58^. CGRP is currently recognized as one of the central migraine mediators, with multiple targets including dural blood vessels^14^, meningeal nerve fibers^12^ and immune cells^11^.

Interestingly, it was proposed, that impaired clearance of CGRP from perivascular space can be a reason for post-traumatic headache after TBI^59^. Surprisingly, here we demonstrate that the release of CGRP in the meninges from K14 mice lacking mLVs is lower compared to WT control group, suggesting that K14 animals could be less prone to trigger migraine attacks via this signalling pathway. Noteworthy, CGRP is vital for lymphatic capillary formation^60^, suggesting a potential positive crosstalk between CGRP signalling and the activation of the meningeal lymphatic system in normal conditions. As local trigeminal nerve fibers are the main source of meningeal CGRP^57,58^, we propose that the lymphatic impairment causes either a reduction of the CGRP storage in trigeminal nerve terminals, or is associated with a decreased innervation of the meninges by CGRP containing nerve fibers^61^.

Meningeal sensory nerve fibers are characterized mainly by nociceptive Aδ and peptidergic C-fibers^62^. While both of them can release CGRP, the latter are considered as the primary source of CGRP^58^. Our analysis indicates that meningeal innervation density and complexity did not differ between the two analysed genotypes, suggesting that the lower CGRP release observed in K14 mice is not the result of a reduced density of meningeal innervation. At the same time, our data exclude the possibility that the lack of lymphatics can promote outgrowth or retraction of meningeal afferents. We can therefore speculate that the observed altered CGRP release in K14 mice rather represents the different neuropeptide content in nerve terminals or is due to a reduced functionality of the CGRP releasing machinery.

Previous studies on migraine patients have mainly focused on the profiling of cytokine from the systemic circulation^50,51,63–66^, whereas the production of cytokines in the meninges have not been fully characterized. Various immune cells can be identified in the meninges, including lymphocytes and mast cells^56^, which can be transported within the lymphatic vessels^67,68^. These cells constitute the primary and major source of multiple pro-inflammatory and anti-inflammatory cytokines. In our ex vivo preparation, pro-inflammatory cytokines (i.e., IL-6, IL-10, MCP-1, TNFα, IL-12p70, and IFNγ) are released and detectable already at basal conditions, confirming the local source of inflammatory stimuli that can trigger migraine. In particular, we demonstrated the presence of the pro-inflammatory cytokines TNFα and IL-6, known to be associated with migraine pathology^51,54,63–66^. Notably, we found that the absence of functional mLVs was associated with increased levels of the pro-inflammatory cytokine MCP-1^69^. MCP-1 is known as the activator of mast cells, provoking release of mast cell-derived components^70^. Remarkably, an increase in MCP-1 was reported in the cerebrospinal fluid of patients during a migraine attack^54^. In contrast to MCP-1 increase, in the preparations from K14 mice we found a decrease in the levels of the pro-inflammatory cytokine IL-12p70, which is typically released from macrophages and dendritic cells^71^. As our hemiskull preparations include also skull bone marrow cells, we cannot exclude that a proportion of the measured cytokines derive from this niche. A recent paper demonstrated the presence of dural channels that provide direct access for the immune cells from skull bone marrow area to the meninges^72^: therefore, we cannot exclude that these skull bone marrow-derived immune cells play a role in the regulation of meningeal inflammation and nociception. This hypothesis and the role of meningeal lymphatics in the control of this trafficking need to be further elucidated.

Extracellular ATP, acting primarily via P2X7 receptors, is a powerful trigger of inflammatory reactions involving different dural immune cells, including mast cells^48,49,73,74^. Consistent with this view, we found that the stimulation of meninges with the P2X7 agonist BzATP enhanced the release of the pro-inflammatory TNFα and Il-10, which are both implicated in migraine pathology^75,76^. However, the released levels of these cytokines did not differ between WT and K14 mice, suggesting that the lack of functional mLVs does not alter the sensitization to migraine.

Finally, our analysis shows that the lack of meningeal lymphatics does not affect the basal activity of meningeal afferents, neither promotes the ATP-induced activation of trigeminovascular nociceptive system. Our data in the K14 model have been confirmed in a different model of mLVs dysfunction, were meningeal lymphatics were ablated in adult mice, therefore validating our main hypothesis that meningeal dysfunction does not affect the migraine pathogenesis. However, a non-significant increase in the ATP-induced nociceptive firing was observed in the meninges from mLVs-ablated mice, suggesting that acquired (but not congenic) modifications of mLVs functionality can differently affect the balance shift in pro-migraine mediators.

Our work, despite providing several lines of evidence supporting our conclusions, leaves however some unresolved questions: e.g. how defects in the meningeal lymphatic system shapes the local immune response, or the specific contribution of the different immune cells as source of inflammatory cytokines in the context of migraine. Moreover, all our data have been acquired using ex vivo meningeal preparations: further studies in in vivo migraine models (e.g., nitroglycerin injection or CSD induction)^77,78^ are needed to confirm and fully characterize the impact of the meningeal lymphatic system on migraine state.

In conclusion, our data show that the developmental deficiency in meningeal lymphatics (as observed in the K14 mice used in this study) results in the local shift in the balance of various humoral factors (i.e., CGRP, MCP-1 and IL12-p70), which are implicated in migraine pathology. On the other side, the observed effects do not result in changes in migraine predisposition: we can, therefore, conclude that per se the lack of a functional meningeal lymphatic system does not support a migraine state*.* Nevertheless, we cannot exclude that the observed balance shift in pro-migraine mediators might contribute to the pathophysiology of the disease in specific conditions predisposing to neuronal sensitization.

## Data Availability

The raw data supporting the conclusions of this manuscript will be made available by the corresponding author, upon reasonable request, to any qualified researcher.

## Abbreviations

WT: Wild type mice
K14: K14-VEGFR3-Ig mice
mLVs: Meningeal lymphatic vessels
